# Mothers’ knowledge and self-reported performance regarding the management of traumatic dental injuries and associated factors: a cross-sectional study

**DOI:** 10.1186/s12887-022-03735-y

**Published:** 2022-11-17

**Authors:** Zahra Momeni, Sanaz Afzalsoltani, Mahmoudreza Moslemzadehasl

**Affiliations:** 1 Community Oral Health Department, School of Dentistry, Alborz University of Medical Sciences, Karaj, Iran; 2 Paediatric Dentistry Department, faculty of dentistry, Tehran medical sciences, Islamic Azad university, Tehran, Iran; 3 Student Research Committee, Alborz University of Medical Sciences, Karaj, Iran

**Keywords:** Tooth injuries, Knowledge, Performance, Self-report, Mothers, Child

## Abstract

**Background:**

Dental trauma is common among children. It has negative effects on a child’s quality of life. Parental knowledge, especially among mothers is an important factor for the long-term success of this emergency. This study aimed to investigate mothers’ knowledge and self-reported performance regarding the management of traumatic dental injuries and associated socio-demographic factors.

**Methods:**

In this cross-sectional study, 277 mothers of children (8–12 years) attending the pediatrics department of Alborz dental school were studied by convenience sampling. The participants completed a valid reliable questionnaire about the management of traumatic dental injuries. The predictor variables were the child’s gender, maternal education and employment status, economic status, and accommodation status. The outcome variables were the mothers’ knowledge about the management of traumatic dental injuries (Eight statements with 10 scores) and self-reported performance including four case scenarios of traumatic dental injury (Five questions with 7 scores). Data were analyzed by T-test or One-way ANOVA to test the within-group changes, Pearson’s correlation coefficient and Linear multiple regression to examine the effect of predictor variables on maternal knowledge and self-reported performance.

**Results:**

The mean score of mothers’ knowledge was 3.43; SD = 1.58 (total score ranged from 0 to 10). The mean score of mothers’ performance was 3.38; SD = 1.2 (total score ranged from 0 to 7). Mothers who work (*p < *0.001) and mothers with high qualifications (*p < *0.001) had higher knowledge. Furthermore, mothers who work (*p* = 0.011), mothers with high qualifications (*p < *0.001), and those who have had previous experience with traumatic dental injuries (*p < *0.001) had higher self-reported performance. The results of multiple linear regression analysis demonstrated the association between mothers’ knowledge with mothers’ education (β = 0.22, *p* = 0.001) and mothers’ employment status (β = 0.15, *p* = 0.017), while there is a relationship between mothers’ self-reported performance and mothers’ education (β = 0.27, *p < *0.001).

**Conclusion:**

Since the means of mothers’ knowledge and performance scores regarding dental trauma management, were less than half of the obtainable scores as well as mothers have a crucial role in the children’s oral health, it is important to increase the mothers’ knowledge and improve their performance in this context. Therefore, running educational programs is necessary for this respect.

**Supplementary Information:**

The online version contains supplementary material available at 10.1186/s12887-022-03735-y.

## Introduction

Traumatic dental injuries with a global prevalence of 6–59%, account for 5% of all physically injuries [1, 2]. Dental trauma has a prevalence of 25% in school-aged children [3]. The prevalence of dental trauma has been reported as 4 to 30%, in developed countries [4]. This type of injury affects the quality of life [5]. Additionally, the biological complications of dental trauma are one of the foremost reasons for paying attention to dental trauma. On the other hand, the cost of treatment can impose a significant economic burden on families and society, and treatment delay can make these costs more [6].

According to the type of injury, dental trauma management is different. These injuries may vary from minor tooth fractures to extensive dentoalveolar damages involving the supporting structures and causing tooth displacement or avulsion. The prognosis after the treatment depends on the fundamental actions taken immediately after the injury. The time interval after the injury and before the initiation of emergency management, and professional treatment, significantly affect the prognosis. Proper and timely management can prevent the aggravation of problems and can have better therapeutic results [2].

As most dental traumatic dental injuries in children often occur at home, the role of parents in managing these events is so important [7]. Parents’ awareness regarding the control and treatment of trauma is crucial for the long-term success of dental trauma. Also, it is a determining factor in valuable improving oral health. Therefore, management of these injuries depends on parental knowledge, especially among mothers, [8] has a significant impact on the long-run lifestyle of their children.

Previous studies that have focus on parents’ knowledge regarding dental trauma in different countries and groups of people show limited knowledge on how to manage dental trauma among lay people, especially mothers [9–11]. A review study by Muraly and et al. [12] emphasized that despite a variety of studies on education and knowledge regarding dental trauma care, the results seem to be consistent, namely that numerous educational procedures for professional caregivers and lay people have failed. It seems to should be focused on laypeople to handle complex cases such as tooth avulsion. Caregivers’ and laypeople’s education are fields in which much research has to be done. The results of a cross-sectional study conducted in Saudi Arabia revealed that only about half of the participants (55.3%) had taken their child to the dentist immediately after a tooth trauma [10]. Similar studies in Iran showed that Iranian mothers’ knowledge of dental trauma management is relatively low [13–15]. For instance, a local qualitative study [16] presented that few Iranian mothers were aware of traumatic dental injuries. They had not acknowledged dental trauma as an oral health problem.

Parents play a crucial role in improvement of treatment and reducing complications of a dental trauma [9, 17]. The outcomes of a dental trauma seriously depend on the appropriate and early management done by non-specialists present at the place of the accident (such as mothers) [18, 19]. As mothers are usually the first point of care for their children, this study aimed to evaluate mothers’ knowledge and self-reported performance regarding traumatic dental injuries management and the associated socio-demographic factors. These factors are important for the quality of life and well-being of children. Evaluation of mothers’ knowledge and understanding in this condition can help healthcare professionals to promote oral health.

## Method

### Study design and population

This study followed the recommendations of the STROBE guidelines adapted for observational studies [20]. This cross-sectional study was conducted in Karaj, Alborz Province, Iran. Alborz Province is northwest of Tehran, one of every 31 provinces of Iran, centered in Karaj. According to the National Census, in 2016 population of Alborz was 2,712,400 which 90.5% lived in urban areas [21].

Based on the following equation, 277 mothers as the minimum required sample size calculated. “n” was the sample size, “α” = (0.05), “SD” standard deviation of oral health knowledge in mothers in the study of Razeghi et al. (1.7), and “d” was the standard error (0.2) [19].

$$n=\frac{{Z}_{1- {\alpha}\!\left/ \! {2}\right.}^{2}\times \text{S}{D}^{2}}{{d}^{2}}$$

Data collection was carried out using a convenience sampling method in the pediatric department of Alborz University of medical sciences from April to June 2019. The mothers of 8- to 12-year-old children who willingly agreed to participate in this study received an anonymous self-administrated questionnaire. There were no other inclusion or exclusion criteria. Mothers were asked to complete the questionnaire while waiting or at the time of their child’s treatment. The questionnaire was completed in approximately 15 min. All the distributed questionnaires were completed and returned in the presence of researcher (MM). Thus, there were no missing data. In the last, a pamphlet regarding dental traumatic management was provided to each mother.

### Study instruments

The data collection tool in the present study was a questionnaire (Additional file 1: Appendix 1) that had previously been standardized and localized in Iranian studies [19]. The questions were collected from similar previous studies [9, 22], the latest guidelines for the management of dental trauma [5, 23], and reference books [3, 24, 25]. A panel of experts (which consisted of two experts in community oral health, one pediatric dentist, and one epidemiologist) assessed the validity of the questions included in the questionnaire [19]. The reliability test was carried out again using the test-retest on 20 research subjects. They were excluded from the main study. The Kappa coefficient was above 70% for different questions.

The questionnaire included demographic information on the child’s gender, education and employment status of mothers, economic status, accommodation status (owner with or without a mortgage, private tenant, and free), and one question about the previous experience of traumatic dental injuries. Years of education as a valid and reliable proxy of social level in Iran [26] was categorized as high school, diploma, and university level.

The mother’s knowledge and self-reported performance regarding the management of dental traumatic injuries were assessed as outcome variables. Eight statements tested knowledge using multiple-choice, or “yes”, “no”, and “I do not know” answers. A score of 0 was given to false or “I do not know”, and each correct answer was scored 1. Two questions had two correct answers. By summing up the scores of eight questions maximum of 10 points can be obtained (range: 0 to 10). The self-reported performance was assessed using four case scenarios of traumatic dental injuries. Each case represented a patient with a certain traumatic dental injury. Overall, five questions tested self-reported performance. One question had three correct answers, so the self-reported performance score of each mother could range from 0 to 7.

### Data analysis

Data were analyzed by the SPSS-22 software (SPSS Inc., Chicago, IL, USA). The characteristics of the research subjects in the form of categorical/dichotomous data were described in frequency (n) and percentage (%). T-test and one-way analysis of variance (ANOVA) was used to compare the mean scores between groups. Pearson’s correlation coefficient (r) investigated associations between knowledge and self-reported performance. In addition, multiple linear regression model was used to adjust the effect of confounding factors by a backward method. The child’s gender, mother’s employment status, mother’s education, economic status, accommodation status, and previous experience of traumatic dental injuries as predictor variables were interred in the regression models. The level of significance was set at *p* < 0.05.

### Research ethics

The participants were informed about the purpose of this study and assured their participation or non-participation would not affect their child’s treatment process. Also, they were ensured about the privacy and confidentiality of information. In addition, data were collected anonymously and stripped of all identities. In case of dissatisfaction, mothers have not entered the study. The Ethics Committee of Alborz University of Medical Sciences approved the study protocol based on verbal informed consent (ethical code: IR.ABZUMS.REC.1398.059). All methods used in this study adhere to the Declaration of Helsinki tenets of 1967.

## Results

### Sample characteristics

The sample consisted of 277 mothers of children aged 8 to 12 years. Out of 277 schoolchildren, 134 were boys (48.4%) and 143 were girls (51.6%) by the mean age of 9.51; SD = 1.42. The mothers were aged between 24 and 47 years (35.33; SD = 4.75).

In terms of education, 178 (64.3%) of mothers claimed to have the university license and 186 (67.2%) fathers. With regard to the employment status, 88 (31.8%) mothers and 13 (4.7%) fathers were unemployed. About the economic situation of 196 participants (70.7%) were reported good and very good. In addition, 153 of the participants (55.2%) were owner and 20.6% (57) mentioned having previous experience of traumatic dental injuries. Table 1 shows the general demographic characteristics of samples.

**Table 1:** The socio-demographic characteristics of the mothers (*n* = 277)

Demographic characteristic		N (%)
Child’s gender		
Boy		134 (48.4)
Girl		143 (51.6)
Mother’s education		
High school		27 (9.7)
Diploma		72 (26)
Universal/Higher education		178 (64.3)
Mother’s employment status		
Employed		189 (68.2)
Unemployed		88 (31.8)
Father’s education		
High school		43 (15.5)
Diploma		48 (17.3)
Universal/Higher education		186 (67.2)
Father’s employment status		
Employed		13 (4.7)
Unemployed		264 (95.3)
Economic status		
very good		7 (2.5)
good		189 (68.2)
poor		60 (21.7)
very poor		4 (1.4)
Do not have idea		17 (6.2)
Accommodation status		
owner with or without mortgage		153 (55.2)
private tenant		118 (42.6)
Free		6 (2.2)
Previous experience of traumatic dental injuries		
Yes		57 (20.6)
No		220 (79.4)

The results showed that mothers who work, and those had qualifications had higher knowledge regarding the management of dental trauma injuries. Regarding self-reported performance, mothers who work, those had qualifications, and those had previous experience of traumatic dental injuries reported better self-reported performance (Table 2). The Pearson correlation coefficient showed that mothers’ knowledge had a positive correlation with mothers’ self-reported performance (r = 0.31, *P* < 0.001).

**Table 2:** Mean score of mothers’ knowledge of the management of traumatic dental injuries at different socio-demographic characteristics (*n* = 277)

	Knowledge			Self-reported performance		
	**Mean**	**SD**	***p*** **-Value**	**Mean**	**SD**	***p*** **-Value**
Child’s gender						
Boy	3.43	1.35	0.1 ^a^	3.47	1.16	0.22 ^a^
Girl	3.43	1.34		3.29	1.23	
Mother’s education						
High school	2.70	1.38	**<0.001** ^b^	2.93	1.17	**<0.001** ^b^
Diploma	2.85	1.50		2.89	1.27	
Universal/Higher education	3.79	1.54		3.65	1.10	
Mother’s employment status						
Employed	3.17	1.58	**<0.001** ^a^	3.64	1.08	**0.011** ^a^
Unemployed	3.99	1.45		3.25	1.23	
Economic status						
Very good	3.71	0.95	0.224 ^b^	3.57	1.40	0.41 ^b^
Good	3.56	1.55		3.39	1.15	
Poor	3.08	1.73		3.48	1.31	
Very poor	2.50	0.29		2.50	0.58	
Do not have idea	3.41	1.50		3.06	1.43	
Accommodation status						
Owner with or without mortgage	3.54	1.61	0.44 ^b^	3.33	1,23	0.32 ^b^
Private tenant	3.31	1.56		3.47	1.17	
Free	3.16	1.33		2.83	1.17	
Previous experience of traumatic dental injuries						
Yes	3.30	1.38	0.16 ^a^	3.02	1.32	**<0.001** ^a^
No	3.50	1.63		3.47	1.16	

Significant results (*p*<0.05) are highlighted in bold

^a^T-test Analysis

^b^one way ANOVA

### Knowledge score of study subjects

The knowledge section consisted of 8 questions. The mean score of the mothers’ knowledge about the management of dental trauma was 3.43, SD (Standard Deviation) = 1.58. The score obtained of the mothers’ knowledge ranged between 0 to 7 points (out of 10 points). The most correct answer in the knowledge section was about “Where is the first place to go after a child’s teeth are traumatized?“ with 78.3% of the correct answer. The least correct answer to the questions in this section was regarding the question “What is the best way to clean an avulsed tooth before replacement?“ with 7.6% of the correct answer. The percent of correct answers to knowledge questions is presented in Table 3.

**Table 3:** Distribution of mothers' favorable answers to knowledge questions regarding traumatic dental injuries management (*n* = 277)

Question	N	%
If a tooth is broken due to injuries, can the broken piece be glued back in place? Answer: Yes	67	24.2
If a baby tooth falls, should it be put back in its place? Answer: No	175	63.2
If a permanent tooth falls, should it be put back in its place? Answer: Yes	102	36.8
Where do you take a child with a dental trauma first? Answer: Dental office	217	78.3
When is the best time for a fallen tooth to be glued back in its place? Answer: Immediately/Less than 30 min	115	41.5
What is the best way to clean a fallen tooth before its replantation? Answer: water	21	7.6
How do you keep the tooth until its replacement? Answer: In milk/Child’s mouth	47	14
What would you do if your child develops a loose tooth due to a trauma? Answer: I will try to replace the tooth	31	11.2

### Self-reported performance score of study subjects

The self-reported performance section consisted of five questions with the help of 4 case scenarios. The mean score of the assessed in this part was 3.38, SD = 1.2 and the lowest and highest scores were 0 and 5, respectively (out of 7 points). The percentage of correct answers to self-reported performance questions is presented in Table 4.

**Table 4:** Distribution of mothers' favorable answers to self-reported performance questions regarding traumatic dental injuries management (*n* = 277)

	n	%
**Case 1**: Your 9-year-old daughter has fallen while was playing in the park. Her upper front tooth is fractured. There are no other injuries.		
Do you think the broken tooth is a baby tooth or permanent tooth? ***Answer***: Permanent tooth	182	65.7
What is the first thing that you would do? ***Answer***: I will find the piece and immediately take my child to a dentist.	191	69
**Case 2**: Your 12-year-old son has fallen while was playing football. His mouth is covered with blood and his upper front tooth is missing. He has no other injuries. What is the best action that you would take? (You can choose more than one option) ***Answer***: I will find the tooth immediately, wash, and replace it in the bone and take the child to a dentist./ I will find the tooth and store it in a suitable storage and take the child to the nearest dentist./ I will find the tooth and give it to the child to store it in his mouth and take him to the nearest dentist.	151	54.5
**Case 3**: Your 10-year-old child has fallen while was playing and lost their consciousness. What is the first action that you would take in this condition? ***Answer***: I will take them to hospital immediately.	235	84.8
**Case 4**: Your 10-year-old child has bumped into their friend while was playing and when you look at them you see that one of their teeth has displaced. They have no other injuries. What would you do in this condition? ***Answer***: I won’t touch the tooth and take them immediately to a dentist.	177	63.9

### Multiple linear regression models

After controlling socio-demographic variables (child’s gender, mother’s employment status, mother’s education, economic status, accommodation status) and previous experience of dental trauma, linear regression analysis showed that mothers’ knowledge score regarding the management of traumatic dental injuries was associated with the mothers’ education (*P* = 0.001, β = 0.22, SE = 0.15) mothers’ employment status (*P* = 0.017, β = 0.15, SE = 0.21). Moreover, mothers’ self-reported performance score was associated with their education (*P* < 0.001, β = 0.27, SE = 0.11).

## Discussion

The present study examined the knowledge and self-reported performance of mothers who had a child aged 8–12 years attending Alborz Dental School regarding the management of dental traumatic injuries and associated factors in 2019. The results provide a basis for improving future interventional programs in the community of mothers. In literature, oral health knowledge was known as a predictor of intention to improve oral health behaviors [27]. In this study, mothers’ knowledge regarding the management of dental trauma injuries was insufficient. The mean score of the participants’ knowledge was almost one-third of the total knowledge score (mean score 3.43 out of a maximum of 10). Their self-reported performance score was moderate (mean score 3.38 out of a maximum 7). Multiple linear regression showed that mothers’ knowledge is influenced by mothers’ education and their employment status with a positive effect. there was an association between self-reported performance and mothers’ education.

The reported insufficient knowledge of mothers in this study is in line with the findings of previous studies [9–11, 13, 14, 28]. A study conducted by Kaul et al. [29] in Kolkata as well as Ozer et al. [30] in Turkey in 2012, was shown that the overall knowledge of parents about emergency trauma management was not satisfactory. A study conducted by Kebriaei et al. in 2020 [13] reported the results are in line with the present study. Irrespective of nationality, the knowledge of mothers regarding dental trauma is inadequate. This finding was similar to a qualitative study that recognized a poor overall knowledge of traumatic dental injuries among Iranian mothers [16]. The difference in studies’ findings can be explained by the diversity in the composition of the samples. The result of the present study and other similar studies all show the lack of knowledge related to dental trauma in mothers and acknowledges the need to provide information in this field in various educational forms in order to improve the knowledge of mothers.

What each person does is more important than what each person knows. Without correct information, no correct decision can be made. Often, this lack of correct decision-making leads to low adherence and even rejection of preventive health measures [31]. Based on the International Association of Dental Traumatology (IADT) guidelines, the best practice in case of permanent tooth avulsion, as a serious dental injury, is immediate replantation of the tooth. It is suggested to find the tooth and wash it for almost 10 s under cold running water and then reposition it. If replantation of the avulsed tooth is not possible, it should be saved in a suitable liquid medium like milk to keep the adhesion ability of the tooth cells as well as alive. The tooth can also be kept inside the lip or cheek and transported to the mouth. It is necessary to emphasize water is not a proper medium [5].

The majority of the participants did not know how to clean and maintain a broken or avulsed tooth to take to the dentist. Few of them mentioned they use water to clean teeth. Almost two in 10 mothers in this study chose milk as a suitable liquid; this finding was similar to Shahnaseri et al. [14], but it was lower than Jabarifard et al. [32] who reported 32.5% of Iranian mothers in Isfahan had chosen milk or saliva correctly as a suitable medium for avulsed tooth transfer. A new study [13] reported mothers’ knowledge in this regard was also insufficient, because only 6.1% and 4.7% of the participants chose milk and saliva as transferring liquid, respectively. Sanu and Utomi reported only 1.1% of Nigerian parents knew that milk is the best choice for transporting an avulsed tooth [33].

The second scenario in the third part of the questionnaire described an avulsed tooth situation. The prognosis of avulsed teeth depends on quick and appropriate treatment, while many studies have reported that mothers are unaware of the possibility of avulsed tooth replacement [8, 14, 34]. Sanu and Utomi reported mother’s ability to replace teeth was poor [33]. In a study was conducted by Al-Sehaibany [10] 46% of mothers supposed, immediately replant the avulsed tooth. As a reason, although an avulsion of a permanent tooth is one of the most serious dental trauma, the frequency is totally low [12]. Therefore, it is necessary to implement various forms of educational interventions is to improve the knowledge of mothers.

In a study conducted by Al-Sehaibany [10], many Saudi mothers (41.6%) stated to protect the avulsed tooth in a proper medium and bring the child to the dentist immediately. The best time for the urgency of permanent tooth replantation is less than 30 min to avoid dehydration of the root surface and injury of the periodontal membrane [5]. 41% of mothers in this study chose the correct answer. 30% had stated immediately and 10% of participants said less than 30 min. These results are in correspondence with the previously published study on Egyptian parents who chose the answer “at any time”. In other words, they did not consider the time factor for replantation [35].

Positive correlations were found between mothers’ knowledge and self-reported performance regarding dental trauma. The mothers’ performance was affected by their unfavorable knowledge in this regard. In confirmation of this issue, we are able to point to the higher performance of mothers who had higher knowledge. Meanwhile, socio-economic, behavioral, and cultural differences can affect knowledge and are of great importance. It seems that the implementation of assorted educational programs like educational classes, and providing printed media or educational software is critical to extending the notice of mothers. Even knowing the effect of dental trauma on the change in the quality of life is of great importance.

Having a previous experience of dental trauma is addressed as an important factor in the management of dental injuries. The previous experience of dental trauma has been introduced as a good predictor of the mother’s knowledge of how to manage a tooth injury [36–39]. Interestingly, this study did not show any association between previous experience and knowledge and the self-reported performance of mothers. A large number of dental injuries are not treated at all. Some people do not visit the dentist and do not receive any education in oral health. Hence, we do not see any changes or progress in their information and performance about a dental trauma.

There are some reasons why people do not seek medical advice following a trauma. It is because they are not aware of the importance of treatment. It could be related to the fact that dental trauma is not considered as serious as other injuries. Dental injuries are not life-threatening, the public usually does not consider them a disease. The economic status of the community and the lack of dental insurance are the other preventive factors to seek medical advice. Therefore the occurrence of trauma has no instructive points for caregivers and those present at the scene. When there is no education and treatment, we will not see an improvement in the performance. Despite this, any type of trauma is worrying therefore individuals will try to get help immediately after a trauma even when they do not know what to do [31]. Several studies have reported interest from people to help a person with an avulsed tooth [12, 36]. There is obviously a sensitive mission to notify the public about the significance of dental trauma, especially complex dental injuries.

The results of linear regression analysis exposed that the mothers’ knowledge was positively associated with their education and employment status. So that mothers who worked and had qualifications had higher knowledge. While multivariate linear regression showed that the performance of participants is affected by mothers’ education. Razeghi et al. [38] showed the relationship between parental knowledge and their employment status was not significant. Mothers with higher education had higher knowledge. This finding is in keeping with previous studies that show education was a significant predictor of knowledge [11, 13, 32, 36]. Opposite to the previous studies [28, 34, 40], in a descriptive-analytical study in Isfahan in 2017, Shahnaseri et al. [14] stated that there was a direct relationship between parental knowledge and their education.

In our society, less-educated women are mostly those who get married and have children at a young age. They don’t have a reliable experience or enough information in any field, including trauma management. Their knowledge is limited to the tips they get from non-specialists. In contrast, women who work and had qualifications have more up-to-date information than non-educated or ill-educated unemployed women. With all this, in spite of the fact, still it seems more education is needed to give to the mothers and caregivers. The authors suggest that it is far better to conduct education at the community and through mass communication tools such as social media so that everyone can have access regardless of their economic or educational status.

Undoubtedly, education will be improved mothers’ knowledge of dental trauma first-aid as well as their overall ability to deal with emergencies. It seems that the low awareness among mothers regarding the management and treatment of dental traumatic injuries has an adverse effect on their performance who feel unable to do so. One of the most important skills in health promotion is problem-solving which creates sustainability in capacity-building approaches [41]. Future oral health programs should focus on enhancing people’s capacity to address personal barriers to realizing optimal oral health.

Since the participants were mothers of children who had attended dental school. It was expected they had more knowledge about oral health. It seems more attention should be paid to emphasizing to dental students how important it is to educate patients about oral hygiene instructions. According to the results of the studies in this field, increasing the awareness of mothers in dealing with dental trauma in children seems to require macro-level planning. It is suggested that careful planning should be considered by health and academic systems to increase awareness of people dealing with dental trauma to seek treatment immediately. On the other hand, to identify the factors affecting the knowledge and self-reported performance of mothers, studies with higher and multi-center sample sizes and if possible, studies with before and after training should be done. It seems, that using useful instructions in educational programs is necessary for dental injury management.

Although there are already some studies that evaluate the mothers’ knowledge and practice regarding traumatic dental injuries, as far as our knowledge, this is the first study measuring the knowledge and self-reported performance of mothers in Karaj by designing some scenarios. Nevertheless, the present study had some limitations. First, opposing longitudinal studies that evaluate temporal relationships, cross-sectional studies lack time. Thus, the present study cannot address changes in conditions over a long period. It can report only a short view of the conditions at a specific time. Second, the sample was restricted to a population of mothers referring to the pediatrics department of Alborz dental school, and the socio-economic context might have influenced the results. As with all cross-sectional studies, there may be some limitations to the generalizability of the findings. Even though it is difficult to generalize the results to all mothers, we believe that similar patterns may be present in other populations.

The most important limitation of the present study was parental incommodity to participate in the study, the participants’ lack of time, and the lack of rewards for respondents. As participants may present different characteristics than those who did not participate, it can lead to underestimating or over outcomes. We were able to overcome these problems (non-response bias or non-participant bias) by explaining the study objectives to the participants and using an anonymous questionnaire as well as reassuring them about not registering personal information. Non-response to some questions was checked by the researcher who asked participants to answer all the questions. Also, an educational pamphlet with content on traumatic dental injury management was provided to mothers.

An aspect that can be considered a strength is using a well-designed questionnaire that assessed knowledge and performance regarding dental trauma management. We used a valid and reliable questionnaire that was designed based on the latest guidelines, reference books, and relevant articles. Also, four scenarios of traumatic dental injuries (case design) were designed to assess mothers’ performance in more realistic situations. Therefore, mothers experienced real situations. However, oral health literacy is an important factor that impacts the mother’s performance regarding oral health problems and should consider in future studies.

The questionnaire was long enough to obtain essential information. The simple language of the questions indicated the design of the questionnaire was suitable. Using a system to score the knowledge provide an opportunity to measure and compare knowledge for different categories. In addition, it would able investigators for future comparisons. Evaluation of mothers’ knowledge and perception would help the healthcare providers to design health promotion programs and promote oral health.

## Conclusion

The present study considers illustrated that the mothers’ knowledge about dental trauma management administration almost is deficient. In addition, the findings expand on the association between mothers’ knowledge regarding the management of traumatic dental injuries and their education and employment status. However, obviously limited performance was observed. It emphasizes the require for fitting strategies to extend oral health promotion intervention for mothers. The authors recommend oral health education ought to make strides in improving knowledge, attitudes, and performance and eliminating barriers to day-by-day oral health care within the community, especially for mothers. We should develop policies and programs to encourage healthy and protective behavior.

## Supplementary Information

**Additional file 1:** **Appendix 1.** Questionnaire.

## Data Availability

The datasets generated and/or analyzed are available from the corresponding author on reasonable request.
