# Supplementary material for: Mothers’ knowledge and self-reported performance regarding the management of traumatic dental injuries and associated factors: a cross-sectional study
Source: BMC Pediatr. 2022 Nov 17;22:665. doi: 10.1186/s12887-022-03735-y (PMC9670471; doi:10.1186/s12887-022-03735-y)
Supplement: Supplementary file 1 — Additional file 1: Appendix 1. Questionnaire. [file 12887_2022_3735_MOESM1_ESM.pdf]

Dear parents,

This questionnaire is to check the Knowledge and performance of the parents of 8- to 12-year-old children regarding the management of dental injuries. The questionnaire is anonymous and all information will remain confidential. Your detailed answers can be very constructive. Your participation in this study is voluntary and your answers do not affect your child's treatment process. Please, complete the questionnaire, attentively.

Date ....

Do you have any previous experience of traumatic dental injuries?

- ☐ Yes
- ☐ No
- ☐ I don't know

1. If a tooth is broken due to injuries, can the broken piece be glued back in place?

- ☐ 1. Yes
- ☐ 2. No
- ☐ 3. I do not Know

2. If a baby tooth falls, should it be put back in its place?

- ☐ 1. Yes
- ☐ 2. No
- ☐ 3. I do not Know

3. If a permanent tooth falls, should it be put back in its place?

- ☐ 1. Yes
- ☐ 2. No
- ☐ 3. I do not Know

4. Where do you take a child with a dental trauma first?

- ☐ 1. Doctor
- ☐ 2. Dental office
- ☐ 3. Hospital emergency department
- ☐ 4. I don't know

5. When is the best time for a fallen tooth to be glued back in its place?

- ☐ 1. Immediately
- ☐ 2. Less than 30 min
- ☐ 3. During some hours
- ☐ 4. After some hours
- ☐ 5. I don't know

6. What is the best way to clean a fallen tooth before its replantation?

- ☐ 1. brush the tooth
- ☐ 2. water
- ☐ 3. water and salt
- ☐ 4. Milk
- ☐ 5. You shouldn't clean the tooth
- ☐ 6. I don't know

7. How do you keep the tooth until its replacement? (You can choose more than one option)

☐ 1. In ice
☐ 2. In Water
☐ 3. In Milk
☐ 4. Child's mouth
☐ 5. Tissue or cloth
☐ 6. Disinfectant solution
☐ 7. Other methods
☐ 8. I don't keep the tooth
☐ 9. I don't know

8. What would you do if your child develops a loose tooth due to a trauma?

☐ 1. I will try to replace the tooth
☐ 2. I let the tooth remain in the child's mouth
☐ 3. I would remove the tooth from the child's mouth

Below, there are 4 scenarios that can lead to dental injuries. After reading each case carefully, answer the questions related to them.

Case 1: Your 9-year-old daughter has fallen while was playing in the park. Her upper front tooth is fractured. There are no other injuries.

Do you think the broken tooth is a baby tooth or permanent tooth?

☐ 1. baby tooth
☐ 2. Permanent tooth
☐ 3. I don't know

What is the first thing that you would do?

☐ 1. If it doesn't hurt or bleed, I won't do anything.
☐ 2. I will find the piece and immediately take my child to a dentist
☐ 3. I don't know what to do.

Case 2: Your 12-year-old son has fallen while was playing football. His mouth is covered with blood and his upper front tooth is missing. He has no other injuries. What is the best action that you would take? (You can choose more than one option)

☐ 1. I will give him a tissue to press hard to stop the bleeding and allow him to rest.
☐ 2. I will find the tooth immediately, wash, and replace it in the bone and take the child to a dentist
☐ 3. I will find the tooth and store it in a suitable storage and take the child to the nearest dentist
☐ 4. I will find the tooth and give it to the child to store it in his mouth and take him to the nearest dentist
☐ 5. I don't know what to do.

Case 3: Your 10-year-old child has fallen while was playing and lost their consciousness. What is the first action that you would take in this condition?

☐ 1. I’ll wake them up and allow them to rest.
☐ 2. I’ll take them to hospital immediately.
☐ 3. I don't know what to do

Case 4: Your 10-year-old child has bumped into their friend while was playing and when you look at them you see that one of their teeth has been displaced. They have no other injuries. What would you do in this condition?

☐ 1. I will replace the tooth with my finger and take them to a dentist, immediately.
☐ 2. I won't touch the tooth and take them immediately to a dentist.
☐ 3. I won't do anything, but if they developed pain later, I'll take them to a dentist.

Demographic information

child's gender
☐ Boy
☐ Girl

Mother's education
☐ High school
☐ Diploma
☐ Universal/Higher education

Mother's occupational status
☐ Unemployed
☐ Employed

Father's education
☐ High school
☐ Diploma
☐ Universal/Higher education

Father's occupational status
☐ Unemployed
☐ Employed

Economic status
☐ very good
☐ good
☐ poor
☐ very poor
☐ Do not have idea

Accommodation status
☐ owner with or without mortgage
☐ private tenant
☐ Free
